# Influence of combined fundamental potentials in a nonlinear vibration energy harvester

**DOI:** 10.1038/srep37292

**Published:** 2016-11-22

**Authors:** Pranay Podder, Dhiman Mallick, Andreas Amann, Saibal Roy

**Affiliations:** 1 Micro-Nano Systems Centre, Tyndall National Institute, Lee Maltings, Dyke Parade, Cork, Ireland; 2 Photonics Centre, Tyndall National Institute, Lee Maltings, Dyke Parade, Cork, Ireland; 3 School of Mathematical Sciences, University College Cork, Cork, Ireland; 4 INSA (Indian National Science Academy) visiting Chair (A. S. Paintal) Professor in Engineering, IACS, Kolkata, India.

## Abstract

Ambient mechanical vibrations have emerged as a viable energy source for low-power wireless sensor nodes aiming the upcoming era of the ‘Internet of Things’. Recently, purposefully induced dynamical nonlinearities have been exploited to widen the frequency spectrum of vibration energy harvesters. Here we investigate some critical inconsistencies between the theoretical formulation and applications of the bistable Duffing nonlinearity in vibration energy harvesting. A novel nonlinear vibration energy harvesting device with the capability to switch amidst individually tunable bistable-quadratic, monostable-quartic and bistable-quartic potentials has been designed and characterized. Our study highlights the fundamentally different large deflection behaviors of the theoretical bistable-quartic Duffing oscillator and the experimentally adapted bistable-quadratic systems, and underlines their implications in the respective spectral responses. The results suggest enhanced performance in the bistable-quartic potential in comparison to others, primarily due to lower potential barrier and higher restoring forces facilitating large amplitude inter-well motion at relatively lower accelerations.

In the foreseeable future the ‘Internet of Things/Everything’ (IoT) is anticipated to impact every major domain of human activity by collecting and analyzing tremendous amount of data from every point of interest through autonomous wireless sensor nodes (WSNs) and thereby enabling intelligent decision-making for a smart environment around human beings. While technological advancements over the last decade have reduced the power consumption of portable electronic devices such as WSNs, data transmitters and implantable medical devices significantly, the problem of reliable energy supply to these devices beyond the limited capability of batteries is yet to be resolved. In this scenario, harvesting electrical energy from ambient mechanical vibrations using vibratory energy harvesters (VEHs) to power the WSNs and portable electronics has been a thriving research topic over the past few years.

The general composition of VEHs often includes a mechanical oscillator and a transducer (electromagnetic, piezoelectric, electrostatic, triboelectric etc.). The conventional linear resonant oscillator based VEHs^1^,^2^ are unable to efficiently convert real-world nonstationary and broadband natural vibrations into electricity due to their characteristically narrow frequency bandwidth. In this context, recently, the nonlinear oscillator based VEH systems have attracted considerable attention due to their inherent capability to improve the off-resonance performance compared to a linear resonant system^3^,^4^,^5^. The nonlinear Duffing equation^6^ forms the theoretical basis of nonlinear vibration energy harvesters, which can take the monostable-quartic (single-well) or the bistable-quartic (double-well) form depending on the nature of the potential energy profile. The monostable-quartic (MQT) potential nonlinearity can be implemented in VEHs through specially designed stretchable spring arms which induce cubic nonlinear restoring force or through nonlinear magnetic levitation force^7^,^8^,^9^,^10^. However, the high energy branch of motion of the quartic systems is largely dependent on the initial conditions and can only be achieved through proper frequency sweep. Furthermore, usually the nonlinear components of the spring force come into effect at large deflection or large limit cycle oscillations (LCO), which can be triggered at relatively large vibrational acceleration.

Physical realization of bistable Duffing oscillator based VEHs has been attempted through repulsive magnetic interaction^11^,^12^,^13^,^14^ or by using buckled cantilever designs^15^,^16^,^17^. However, in practice, the bistability induced through repulsive magnetic interaction are not bistable-quartic (BQT), but rather bistable-quadratic (BQD) in nature, where the oscillators follow quadratic potential profile at large deflections. This fundamental difference in the large deflection behaviors of the theoretically defined bistable-quartic Duffing VEHs and the experimentally deployed bistable-quadratic VEHs lead to substantially different frequency spectrum and energy harvesting performance. This work explicitly highlights these differences and their effects through numerical simulation and experimental validation, which has long been overlooked in the vibration energy harvesting literature. The buckled beam induced bistability, on the other hand, produces quartic potentials due to stretching deformation leading to bistable-quartic (BQT) system^15^,^16^,^17^. However, the buckled beam based BQT systems also suffer from very small oscillation amplitude, limiting the power output severely^18^, and the lack of dynamical adjustability or tunability of the quartic potential, which is governed largely by the mechanical properties of the beam.

In this paper we introduce a novel VEH device topology where magnetic repulsion induced bistability is combined with stretching induced quartic potential in a single design with a tunable bistable-quartic (BQT) potential profile to enhance the performance across the vibrational spectrum. Furthermore, this device topology allows activation of the individual bistable-quadratic (BQD) or monostable-quartic (MQT) nonlinearities, or their simultaneous activation leading to a bistable-quartic (BQT) nonlinear potential, while other parameters remain unaffected. Therefore, this device topology also offers the unique opportunity to make proper unbiased comparison of the performances of VEHs in different nonlinear potential energy profiles. The proposed VEH system induces bistability through repulsive interaction of permanent magnets and administers quartic potentials by stretching of clamped-constrained beams, which exhibits substantially larger amplitude oscillation across the double potential wells in comparison to the clamped-clamped buckled beam based BQT devices. The numerical simulation and experimental results show significant improvement in spectral response of the BQT device in comparison to the BQD and MQT, particularly in the lower frequency domain of the vibrational excitation. The enhanced performance of the BQT device in the lower frequency domain is advantageous in real application scenario since most of the naturally occurring mechanical vibration energy is available in the lower range of the spectrum. Comparison of the harmonic frequency responses of the VEH systems reveals that the BQT device efficiently combines the beneficial features of the BQD (large amplitude oscillation at the lower end of spectrum) and MQT (large harmonic oscillation at high frequencies) devices to generate higher overall power across the vibrational spectrum. In band limited random vibration scenario, the BQT VEH produces more power over broader frequency range, resulting into larger average power than the BQD and MQT VEHs.

## Results

### Bistable-quartic nonlinear energy harvester

**Figure 1:**
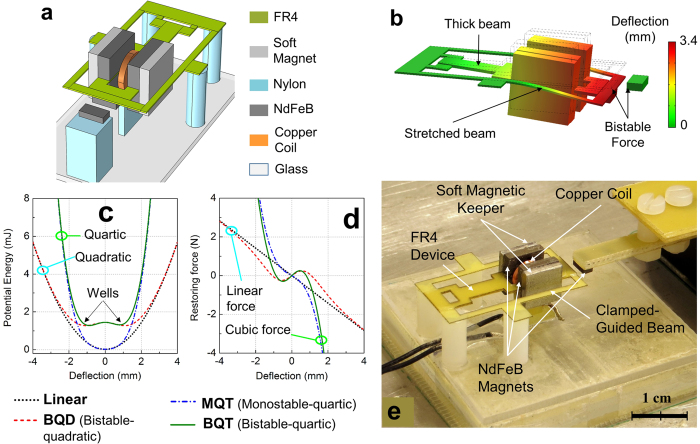
(**a**) Vibrational energy harvester device structure. (**b**) Simulated deflection of the VEH device. The clamped-constrained sections of the oscillator structure get stretched at larger deflections. (**c**) Potential energy and (**d**) Restoring force plots for the linear, monostable-quartic (MQT), bistable-quadratic (BQD), and bistable-quartic (BQT) configurations. MQT follows linear and BQT follows bistable potential at small deflections. At large deflections both MQT and BQT follow quartic potential. (**e**) Side view of the experimental set up. The external repulsive magnet is mounted on a micro-positioning stage for precise control of gap distance.

An important aspect of this work is to understand the fundamental differences in the three types of nonlinear VEHs and a comparative study of their performances under identical excitation conditions. In order to compare the relative performances of the different nonlinear VEHs, we have engineered a VEH system that can be transformed into BQD, BQT or MQT by simple alterations, while keeping the equivalent mass and underlying linear stiffness constant. The proposed bistable-quartic (BQT) VEH system, comprising a laser micro-machined FR4 device structure, miniaturized wire-wound copper coil and NdFeB permanent magnets is shown in Fig. 1(a). The pair of repulsively positioned magnets at the vertically movable constrained end of the device structure produces the bistability, whereas the stretching of a pair of clamped-constrained FR4 cantilevers arranged on either sides of the transducing magnet-coil assembly contributes to the cubic nonlinear stiffness (Fig. 1(b)). The BQT system can be transformed into a monostable-quartic (MQT) system by increasing the gap between repulsive magnets sufficiently or a bistable-quadratic (BQD) system by removing the pair of clamped-constrained beams, while keeping the linear stiffness unaffected. The resulting simulated potential energy and restoring force profiles of the different nonlinear VEH configurations (MQT, BQD and BQT) are shown in Fig. 1(c,d), where the corresponding linear potential and stiffness are also shown as reference. It can be observed that the BQT potential energy and restoring force profiles are considerably modified including two favorable features from that of the BQD and MQT systems. The first is the reduced relative depth of the bistable potential wells with respect to the potential crest leading to a potential energy profile with relatively even or flat bottom. The second is the higher restoring force due to the steeply rising cubic nonlinear force when the oscillator is further away from the equilibrium position. These beneficial factors enable the BQT system to swing from one bistable potential well to the other even at low vibrational accelerations and perform large amplitude inter-well oscillation, leading to superior performance of the BQT system over the others. The fabricated BQT VEH device is shown in Fig. 1(e).

In the context of VEH systems, bistable nonlinearity can be realized by repulsive magnetic force between a pair of permanent magnets, or through buckling of clamped-clamped cantilevers under critical buckling load. At small deflections, the oscillators in both cases experience two potential wells and negative stiffness on either sides of the equilibrium point. At large deflections, however, the effect of magnetic repulsion is negligible and the oscillator experiences a quadratic potential due to the linear stiffness of the cantilever resulting a bistable-quadratic (BQD) oscillator. On the other hand, the buckled beam based bistable oscillator experiences stretching under deflections and experiences quartic potential producing a bistable-quartic (BQT) oscillator. However, the buckling based VEH suffers from very small oscillation amplitude and proportionally small harvested power compared to the magnetic repulsion induced bistability. We overcome the small amplitude oscillation in buckling induced bistable-quartic VEHs in the proposed BQT VEH system by introducing bistability through magnetic repulsion in a clamped-constrained beam architecture. The proposed BQT nonlinear mechanism can be further modified by bringing in even more stable states (tristable, multistable etc.), degrees of freedom (multimode vibration) and miniaturized to the micro-scale.

### Analytical description of bistable-quartic (BQT) nonlinearity

The fundamental distinction among different nonlinear mechanisms in the BQD, MQT and BQT systems can be analytically described through the generalized equation of the Duffing potential energy (*U*_*D*_(*y*)) and the corresponding spring reaction force (*F*_*D*_(*y*)) as given by^3^,^19^,


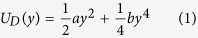



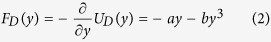


where *a* and *b* are independent parameters and *y* represents the displacement or deflection of the oscillator. Depending upon the values of *a* and *b* the potential energy function can represent either a linear (*a* > 0, *b* = 0), monostable-quartic (*a* > 0, *b* > 0) or bistable-quartic (*a* < 0, *b* > 0) potential energy profile^19^. The linear oscillator follows a quadratic potential and linear stiffness profile for the entire range of displacements. In the vicinity of the initial equilibrium 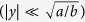 position, the potential energy (*U*_*D*_(*y*)) and restoring force (*F*_*D*_(*y*)) profiles of the MQT oscillator is approximately similar to that of the linear oscillator. However, within the same region, (*U*_*D*_(*y*)) and (*F*_*D*_(*y*)) for the BQT oscillator represents double-well potential and negative stiffness. For large displacements 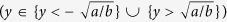 both the theoretical MQT and BQT oscillators produces quartic potential and cubic restoring force profiles, which are significantly steep in comparison to the potential and restoring force of the linear and BQD oscillators.

One of the most widely exploited method of incorporation of bistable nonlinearity in VEH systems is by using repulsively positioned magnets such that pitchfork bifurcation is induced as the distance between the magnets is reduced below a critical limit^20^. While the dynamical behavior of such functional bistable oscillators are governed by the relative heights of the potential barrier and wells near the small deflection region, it is dominated by the regular linear quadratic potential at large deflections where the repulsive interactions between the magnets is negligible. This configuration of bistable and quadratic potentials produces interesting dynamical phenomena e.g. hysteresis in frequency sweep responses, cross-well jump and chaotic oscillations etc. However, it should be noted while the small deflection behavior in both the analytically described BQT oscillator and experimentally realized BQD oscillators are essentially similar, the large deflection behaviors are substantially different. The large deflection behavior in the BQT oscillator is influenced by the cubic stiffness, while that for the BQD oscillator is governed by the linear stiffness. This difference in the large deflection behavior of the theoretically investigated BQT oscillator and the experimentally deployed BQD oscillator leads to remarkably different frequency responses and energy harvesting capabilities, which has not been appreciated in previous works. In fact, many of the reported works use Duffing BQT oscillator in numerical simulations as the quartic term arises from the Taylor series expansion of magnetic repulsion potential in small deflection regime, but use BQD oscillator in experiments since it naturally occurs when an oscillator with quadratic potential is made bistable through repulsive magnetic interactions.

**Figure 2:**
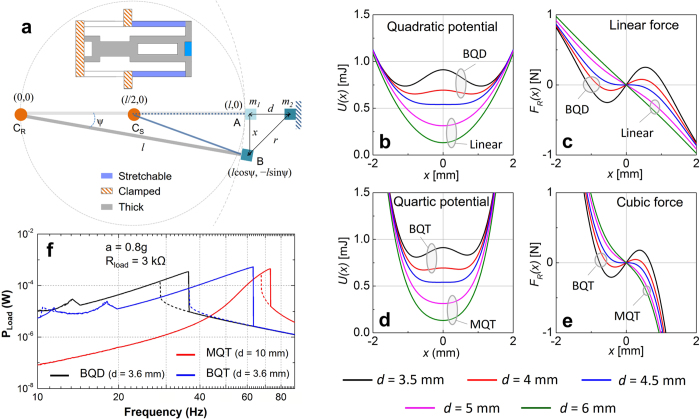
(**a**) Schematic model of the BQT oscillator. The thick, stretchable and clamped sections of the spring structure are also identified. Variation of potential energy function [*U(x)*] and restoring force [*F*_*R*_*(x)*] for different values of *d*. **(b)** Quadratic potential, **(c)** Quadratic force, **(d)** Quartic potential, **(e)** Quartic force. **(f)** Numerically simulated frequency responses for the BQD (bistable-quadratic), MQT (monostable-quartic) and BQT (bistable-quartic) VEHs. The solid and dashed lines represent the forward sweep response and reverse sweep responses respectively.

In order to understand the principal source of nonlinear spring reaction force in our BQT system, let us consider the simplified schematic model as in Fig. 2(a). The spring structure consists of a thick section which has a fixed length *l*, and a thin section of length *l*/2, which is assumed to be stretchable under longitudinal strain. The thick section (section C_R_A of length *l* and thickness *t*_*R*_) and the stretchable thin section (section C_S_A of length *l*/2 and thickness *t*_*S*_, *t*_*R*_ ≃ 2.5 *t*_*S*_) of the beam are conjoined at the movable end A. The thick section and the stretchable sections of the structure are clamped at the other end at different points (C_R_ and C_S_ respectively) such that the stretchable part is approximately half the length of the thick part. Under sufficient vibrational excitation, the movable conjoined end is subject to vertical deflection (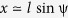 for small deflections), and is constrained to move along the conjoined end of the thick beam. Therefore, the conjoined end of the thin stretchable beam is also constrained to move along with the thick beam, which exerts longitudinal stretching strain in the stretchable section of the beam. The longitudinal stretching strain generates a nonlinear cubic force-displacement relationship or quartic potential condition in the device structure. Considering the angular deflection of the thick section to be ψ, the stretched length of the stretchable beam (length *l*/2) can be estimated as,


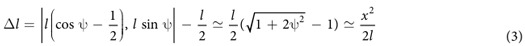


where the approximations are true for small angle ψ. Then, for an elastic beam (of length *l*/2) within the limit of elasticity, the energy stored due to the stretching component of strain can be expressed as^21^,^22^,


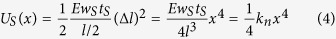


where *E* is the modulus of elasticity (Young’s modulus) of FR4 material, *w*_*S*_ and *t*_*S*_ are the width and thickness of the stretchable beams respectively, and *k*_*n*_ represents the nonlinear spring coefficient due to stretching of the beams. The total strain energy due to bending of the thick and stretchable beam sections can be approximated by considering the Eular-Bernoulli beam formulation for linear elastic solids as^9^,^22^,


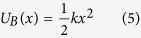


where *k* is the total linearized stiffness coefficient due to bending of the beam. The magnetic interaction potential energy (*U*_*m*_(*x*)) due to the repulsive arrangement can be determined by considering dipole-dipole interaction between the tip magnets as^14^,


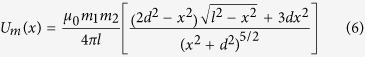


where *μ*_*0*_ is the permeability of air, *m*_*1*_ and *m*_*2*_ are the magnetic dipole moments of the repulsively oriented magnet pair, *d* is the horizontal gap distance between the centers of the magnets when they are anti-parallel and in front of each other and *r* is the distance between the centers of the oppositely polarized tip magnet and external magnet at the deflected position B (Fig. 2(a)). The intensity of repulsive magnetic force on the tip of the cantilever can be controlled by altering *d*, the gap between the tip magnet and the externally positioned magnet. Therefore, the magnetic interaction potential and its contribution to the total mechanical potential energy and restoring force can also be modified by adjusting *d*.

The total elastic potential energy (*U*(*x*)) and spring reaction force (*F*_*R*_(*x*)) taking into consideration the effects of bending, stretching and repulsive magnetic interaction simultaneously can be expressed as,


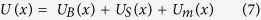



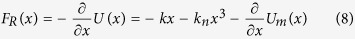


The proposed BQT system allows for the manipulation of quartic and bistable potentials independently of each other. The effect of nonlinear force contribution due to magnetic repulsion induced bistable nonlinearity and stretching induced cubic force nonlinearity on the potential energy (*U*(*x*)) and restoring force (*F*_*R*_(*x*)) are illustrated in Fig. 2(b–e) for different gap (*d*) distances between the repulsive magnets. In the absence of the clamped-constrained cantilevers, the oscillator is subject to a quadratic potential or linear force (Fig. 2(b,c)). On the other hand, the inclusion of the clamped-constrained cantilevers subjects the oscillator to a quartic potential or cubic force (Fig. 2(d,e)). The repulsive interaction between the magnets is negligible for larger gap (*d* ≥ 5 mm), resulting in monostable quadratic (Fig. 2(b)) or quartic (Fig. 2(d)) potentials, and linear (Fig. 2(c)) or cubic (Fig. 2(e)) stiffness profiles. For smaller gaps (*d* ≤ 4.5 mm), the repulsive interaction becomes significant and a bistable condition is produced due to a pitch-fork bifurcation. The resulting potential energy profiles exhibit two potential energy minima (Fig. 2(b,d)) and negative stiffness (Fig. 2(c,e)), characteristic of bistable systems.

In the BQD configuration [Fig. 2(b,c)], the potential function is quadratic and the stiffness is almost linear at large deflections (*x* > 2 mm). Contrastingly, the BQT configuration at similarly large deflections follows a quartic Duffing potential and cubic nonlinear stiffness. The most advantageous consequences of designing a BQT oscillator by combining double-well potentials and cubic stiffness is the modest decrease in the relative height of the potential crest (Fig. 2(d)) with respect to the potential wells and higher restoring force (Fig. 2(e)) at large deflections. The resulting reduced potential barrier can be traversed by the oscillator at lower amplitude vibrations, triggering large amplitude inter-well motion that produces higher energy.

Taking into account the bistable-quartic nonlinearity produced by combining the cubic stiffness and bistable double-well potentials, the complete dynamical system equation can be expressed as,


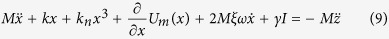


where *M* is the equivalent mass of the system, *ξ* is the mechanical damping coefficient, *γ* is the electromagnetic coupling factor, *I* is the current induced in the coil and, 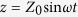 is the displacement due to external vibration. The angular frequency of the external vibration is denoted by *ω*. If the total resistive load combining the coil resistance and the load resistance in the electrical circuit is denoted by *R*, the electrical circuit can be represented as^14^,^17^,


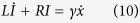


where *L* denotes the inductance of the coil. Eqs (9) and (10) are solved numerically using explicit fourth-order Runge-Kutta method in MATAB to obtain the voltage and power generated in the system.

The numerical simulation results (Fig. 2(f)) exhibit much higher power (~10 times) in the lower frequency domain (10–30 Hz) for the BQD and BQT systems in comparison to the MQT VEH. Moreover, in the high frequency regime (>60 Hz), the BQT system exhibits high peak power frequency and high peak power level almost similar to the MQT system. Therefore, the proposed BQT system essentially combines the beneficial features of the BQD (higher power in low frequencies) and MQT (high peak power in high frequencies) systems to provide a generally improved response. Subsequently, the simulation results are validated against the test results of the fabricated prototypes of bistable-quadratic, monostable-quartic and bistable-quartic VEH devices.

### Experimental optimization of electrical load

**Figure 3:**
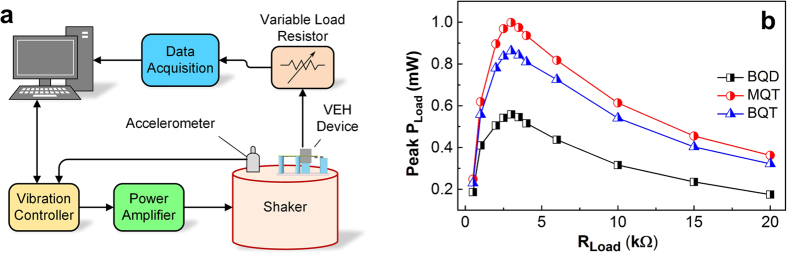
(**a**) Vibration test setup comprising computer controlled electromagnetic shaker, accelerometer, VEH device and digital storage oscilloscope. **(b)** Variation of peak load power (Peak *P*_*Load*_) with load resistance (*R*_*Load*_) for the BQD, MQT and combined BQT configurations at 0.8 g acceleration.

The experimental test set up consisting of a computer controlled shaker, accelerometer and oscilloscope used for the electrical characterization of the VEHs is shown in Fig. 3(a). In the first set of experiments, the optimal resistive load for all the nonlinear (BQD, MQT and BQT) VEH configurations are determined. The pair of clamped-constrained beams in the BQT device structure was discarded in the BQD configuration to avoid the stretching effect, and relatively thicker supportive beam is used to ensure that the linearized resonance frequency for all the configurations remain nearly same (~45 Hz). The excitation frequency in each configuration is ramped up to the jump frequency in the forward sweep and is held constant at a value proximal to the jump frequency. It is shown in Fig. 3(b) that for all the configurations, the peak load power reaches the maximum values at 3 kΩ, the optimum load resistance of the system, which indicate that the effective electrical damping is independent of the mechanical damping in the system. Henceforth we use the optimum 3 kΩ load resistance for all the subsequent experiments on all nonlinear VEH configurations. The forward sweep jump frequencies (*f*_*Peak*_) at 0.8 g acceleration for the BQD, MQT and BQT combined configurations are 38.2 Hz, 73.35 Hz and 65.06 Hz respectively, and the peak load power (peak *P*_*Load*_) at the same acceleration for these three device configurations are 0.487 mW, 1.073 mW and 0.835 mW respectively.

### Response to harmonic frequency sweep excitations

**Figure 4:**
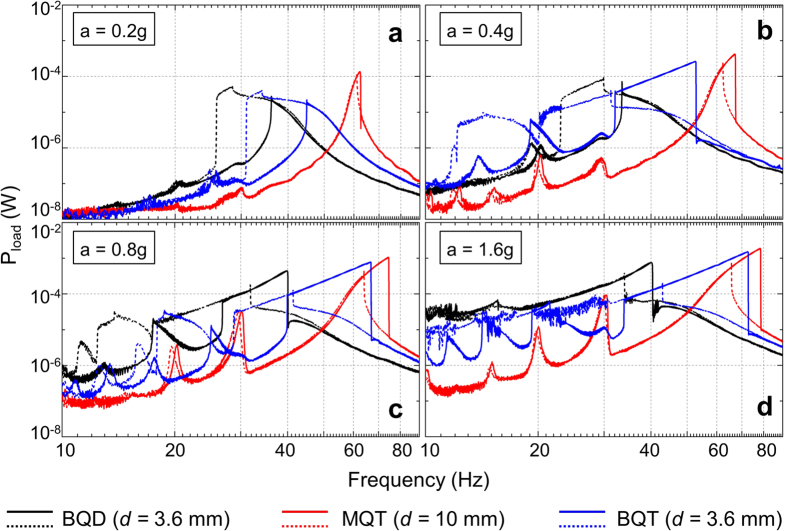
Experimental frequency responses for the BQD (*d* = 3.6 mm), MQT (*d* = 10 mm), and BQT (*d* = 3.6 mm) configurations at different accelerations. Solid lines represent the forward sweep response [10–90 Hz] and dashed lines denote the reverse sweep response [90–10 Hz]. At low acceleration (0.2 g) BQD and BQT VEHs are confined in a single potential well and exhibit softening response leaning towards the left. At higher accelerations (0.4 g, 0.8 g, 1.6 g) the BQD and BQT configurations perform consistent inter-well jumps in the high-energy branch. At 1.6 g acceleration, both BQD and BQT VEHs perform chaotic inter-well motion in the low (10–30 Hz) frequencies. The MQT device produces hardening frequency response leaning towards the right and super harmonic peaks in the low (10–30 Hz) frequencies.

The harmonic frequency responses of the different nonlinear VEH configurations were obtained from vibrational frequency sweeps in the forward (10–90 Hz) and reverse (90–10 Hz) directions while keeping the acceleration constant. The frequency response plots for the BQD, MQT and BQT configurations at geometrically increasing vibrational accelerations (0.2 g, 0.4 g, 0.8 g and 1.6 g) are illustrated in Fig. 4. While the gap between the repulsively positioned magnets is fixed at *d* = 10 mm for the MQT configuration, it is set at *d* = 3.6 mm for the BQD and BQT configurations. At 0.2 g, the BQD and BQT VEHs produces hysteretic frequency responses tilted towards the left with softening characteristics^23^, which is typical of low amplitude intra-well oscillation. This type of small limit-cycle intra-well oscillation indicates that 0.2 g vibrational acceleration is inadequate for the bistable oscillators to overcome the potential barrier and the oscillator essentially remain confined within a potential well. Contrastingly, the MQT VEH produces mildly hysteretic hardening response^23^ by oscillating with small amplitude near the bottom of the single potential well (Fig. 2(d)). As the vibrational acceleration is increased in geometric progression (0.4 g, 0.8 g), the BQD and BQT VEHs eventually escape the potential well under suitable initial conditions and perform large amplitude (large limit-cycle) inter-well motions producing broadband hardening response. The MQT oscillator also produces hardening response at higher accelerations, albeit over a narrower frequency range. Additionally, all the nonlinear VEH configurations exhibit super-harmonic peaks at lower frequencies which become more prominent with increasing accelerations. At 1.6 g acceleration, the bistable VEHs generate higher power in the low frequency regime (10–30 Hz) than the monostable one by performing large amplitude chaotic inter-well oscillations. In the high frequency regime (60–80 Hz) the BQT VEH produces large power similar to the MQT device, which is much higher than that from the BQD VEH. Therefore, it is experimentally validated that the BQT VEH combines the beneficial features of the BQD and MQT VEHs in the low and high frequency domains respectively to harvest higher energy over the entire frequency range.

A figure of merit (FOM) for the comparison of the relative performances of the different VEHs should be able to determine the probability of generating sufficient power to meet certain threshold conditions over the entire frequency range of interest. Since the perception of frequency naturally occur in the logarithmic scale (e.g. pitch of music, color of light, mechanical vibration etc.), the logarithm of vibrational frequency should be incorporated in the FOM expressed as,


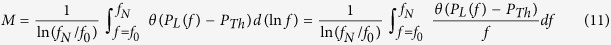


where *f*_*0*_ and *f*_*N*_ are the lower and upper limits of the frequency and *θ* is the Heaviside function defined as,


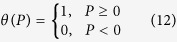


where *P* is the difference between load power *P*_*L*_(*f*) and threshold power *P*_*Th*_ given by,


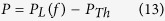


In other words, the function *M* estimates the probability of the load power *P*_*L*_ being higher than the threshold level *P*_*Th*_ over the frequency range *f* = [*f*_*0*_, *f*_*N*_]. The threshold power *P*_*Th*_ is determined by the minimum requirements of the power management electronics of the device driven by the VEH and typically ranges from very low (~1 μW) to very high (~hundreds of μW) values. Since frequencies in our experiments are sampled in equal steps over the entire range, *M* can be approximated as,


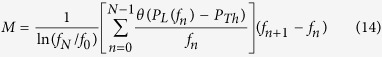


where *f*_*n*_ is the n-th frequency value.

**Figure 5:**
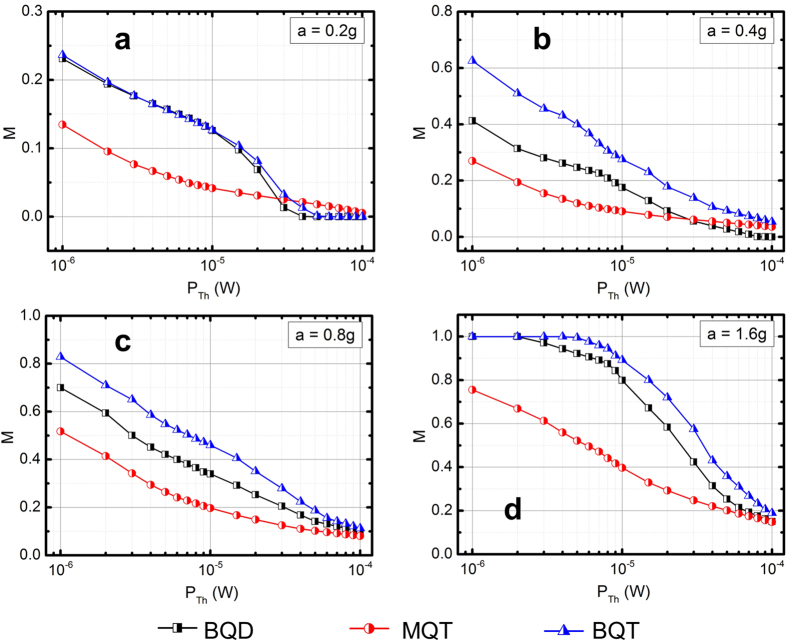
Variation of *M* (normalized probability for *P*_*Load*_ ≥ *P*_*Th*_ over the frequency sweep) with threshold power (*P*_*Th*_) for BQD, MQT and BQT VEH configurations.

The variation of *M* over different values of logarithmically varying threshold power at geometrically increasing accelerations is shown in Fig. 5, where *M* increases with increasing acceleration for all the nonlinear VEH configurations. On the other hand, as the required *P*_*Th*_ is increased logarithmically at fixed accelerations, a smaller segment of the frequency response meets the required power level leading to decreasing the probability *M*. The Fig. 5 shows that for all acceleration values the probability of the BQT VEH to be able to generate load power beyond the threshold value is higher than both BQD and MQT VEHs. The higher probability of harvesting larger than threshold power in the BQT VEH system originates from the combined effect of large amplitude inter-well motion at low frequencies (feature of BQD) and nonlinear cubic stiffness induced large amplitude motion (feature of MQT) in the BQT system.

Another FOM in terms of energy generating capabilities can be defined as the normalized power integral (*NPI*) which computes the amount of usable load power above the threshold power level given by,


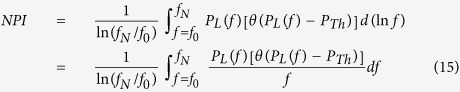


which, due to equidistant frequency steps, can be approximated as,


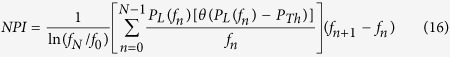


In other words, the *NPI* determines the total usable load power generated above the threshold power level. This FOM appreciates the efficacy of a VEH system in most practicable situations where the generated power is stored in charge storage devices (super-capacitors or rechargeable batteries) after proper conditioning.

**Figure 6:**
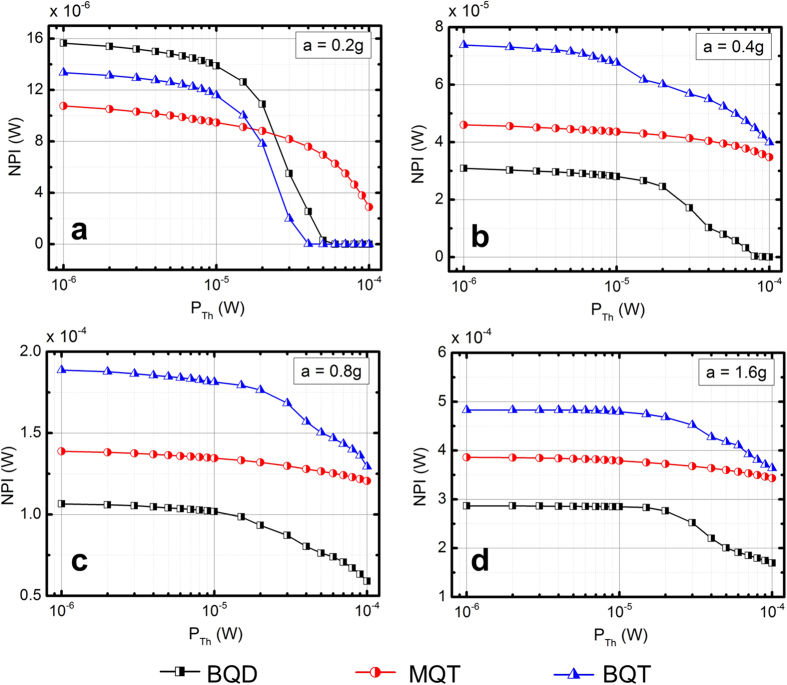
Variation of *NPI* (normalized power density over the frequency sweep) with threshold power (P_Th_) for BQD, MQT and BQT VEH configurations.

The variation of *NPI* over logarithmically varying *P*_*Th*_ are shown in Fig. 6, where the *NPI* is increasing with the geometrically increasing accelerations. At a fixed acceleration, however, the *NPI* gradually decreases as the *P*_*Th*_ is logarithmically increased. This is a result of decreasing the probability of the load power *P*_*L*_ being higher than the threshold *P*_*Th*_ as it is gradually increased. At low acceleration (*a* = 0.2 g) the *NPI* for both BQD and BQT are larger than that of MQT when the threshold *P*_*Th*_ is less than 20 μW. This is a manifestation of the fact that the BQD and BQT harmonic frequency responses tend to be above *P*_*Th*_ at more segments of the response than the MQT VEH. As *P*_*Th*_ is increased gradually beyond 20 μW, the frequency response is unable to cross the threshold level after *P*_*Th*_ = 50 μW and results into zero *NPI*. At higher accelerations, (Fig. 6(b–d)) the *NPI* levels for BQT is higher than that for both BQD and BQT, which indicates higher power harvesting capability for the BQT VEH. This enhancement of *NPI* can be attributed to the fact that the BQT configuration leads to higher power in the low frequency regime (due to bistable nonlinearity), while maintaining the high energy branch of the frequency response similar to MQT (due to quartic potential) in the high frequency regime.

### Band-limited random frequency response

**Figure 7:**
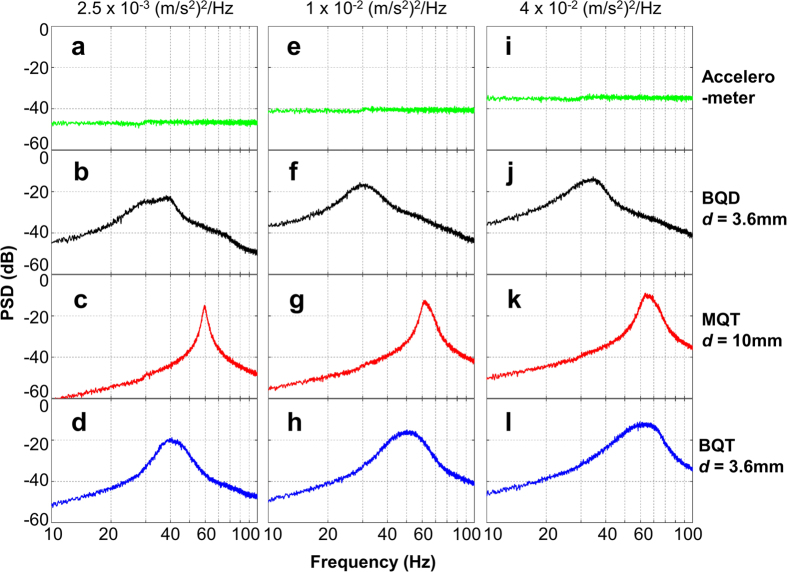
Power spectral density (PSD) vs frequency plots for the BQD, MQT and BQT configurations under band limited (10–110 Hz) random vibrations of 2.5 × 10^−3^ (m/s^2^)^2^/Hz, 1 × 10^−2^ (m/s^2^)^2^/Hz, and 4 × 10^−2^ (m/s^2^)^2^/Hz average PSD.

The performances of all the different configurations of the proposed VEH device in random vibration environment are evaluated in applied bandlimited (10 Hz ‒ 110 Hz) random vibrations with low (2.5 × 10^−3^ (m/s^2^)^2^/Hz, or 0.5 m/s^2^ RMS), medium (1 × 10^−2^ (m/s^2^)^2^/Hz, or 1 m/s^2^ RMS), and high (4 × 10^−2^ (m/s^2^)^2^ /Hz, or 2 m/s^2^ RMS) accelerations for 500 seconds. The resulting PSDs of the input vibrational excitations reveal an uniform distribution of vibrational energy across the spectrum (Fig. 7(a,e,i)). The resulting PSDs of the BQD device produce relatively broadened responses with a peak between 30–40 Hz for all accelerations (Fig. 7(b,f,j)). At all acceleration levels, the MQT variant of the device produces steep peaks near 60 Hz (Fig. 7(c,g,k)), which implies higher peak power but reduced low frequency responses. Contrastingly, in the BQT configuration, the peak is flattened with higher power in the lower frequency domain for all accelerations (Fig. 7(d,h,l)), implying more uniformly distributed energy harvesting capabilities across the spectrum. This is attributable to the higher nonlinear restoring force and lowering of the bistable potential barrier height (Fig. 2(c,d)) due to incorporation of quartic potential, which enable the oscillator to perform inter-well jump at all vibrational accelerations.

**Figure 8:**
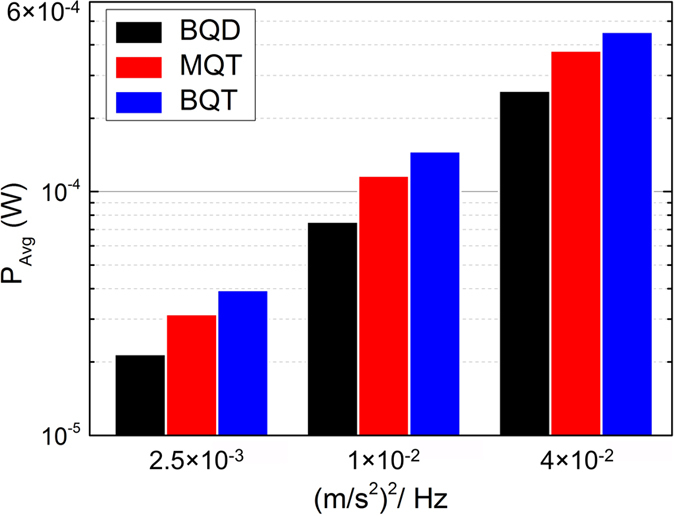
Comparison of average harvested power (P_Avg_) for the BQD, MQT and BQT configurations subject to band limited (10-110 Hz) random vibrations of 2.5 × 10^−3^ (m/s^2^)^2^/Hz, 1 × 10^−2^ (m/s^2^)^2^/Hz, and 4 × 10^−2^ (m/s^2^)^2^/Hz average PSD.

Unsurprisingly, the peak power generated by the BQT VEH is smaller in comparison to the MQT at all accelerations, similar to the observation made previously in case of harmonic excitation (Fig. 3(b)). However, comparisons of the average load power (*P*_*Avg*_) over the entire time series of the random vibrations exhibit that the *P*_*Avg*_ for the BQT device is higher than that of the BQD and MQT configurations (Fig. 8). The broader frequency spectrum and lower peak power and still higher average power in the BQT configuration implies that the harvested energy is spread out more uniformly over the frequency range even in random vibrations.

## Discussion

In many vibration energy harvesting literature, the use of nonlinear bistable-quartic Duffing oscillator based theoretical framework and the practical implementation using magnetic repulsion induced bistable-quadratic nonlinear device leads to critical inconsistencies in the large deflection characteristics and performances of the reported VEHs. We have demonstrated through a specially designed BQT oscillator based VEH that the fundamentally different large deformation behavior leads to significant enhancement in the performance of the BQT device in comparison to both BQD and MQT devices. The proposed novel BQT VEH prototype is fabricated using laser micro-machined spring structure where quartic potential is induced through a pair of clamped-constrained cantilevers and bistability is introduced by the repulsive interaction between discrete permanent magnets and transduction is incorporated electromagnetically. The ability to manipulate the magnetically induced bistability and stretching induced quartic potential independently yields the opportunity for comparative study of the nonlinear effects on the performances of the BQD, BQT and MQT VEHs.

The numerical simulations and experimental harmonic frequency sweep responses reveal that the beneficial features of the BQD (large amplitude chaotic oscillations at low frequency) and MQT (large amplitude harmonic oscillation in the high energy branch at high frequencies) devices are combined in the BQT device, leading to superior performance. Both the probability of generating at least the threshold power and the temporal average of generated power are higher in the BQT device than BQD and MQT devices at all accelerations. Additionally, under bandlimited random vibration the BQT device attains a broader power spectral density and spectral average power in comparison to the other nonlinear systems. These performance enhancements are attributable to the moderate reduction in bistable potential barrier height and higher restoring force at large deflections due to coupling of magnetic repulsion induced bistability into a stretching induced quartic potential system. We demonstrated through numerical simulations and experimental results that the favorable features of nonlinear BQD and MQT systems can be integrated in a combined bistable-quartic (BQT) potential system to enhance the overall performance over the entire vibrational frequency spectrum. The proposed BQT nonlinear device topology could be further modified for implementation in other transduction (piezoelectric, electrostatic, triboelectric etc.) mechanisms for different wideband practical application scenario and miniaturized to the micro-scale. The technique of exploiting the advantages of different nonlinear mechanisms by combining them in a single system could be extended even further towards sensors and accelerometer applications where large amplitude oscillation over broad spectrum of vibrations is necessary.

## Methods

### Numerical simulation method

The dynamical equation of the bistable-quartic combined nonlinear system was solved numerically using 4^th^ order Runge-Kutta method in Matlab. The equivalent mass (*M*), magnetic dipole moments (*m*_*1*_*, m*_*2*_), mechanical damping factor (*D*), total circuit resistance (*R*) and coil inductance (*L*) were measured. While the equations (4) and (5) explains the mechanisms of the quartic and quadratic potentials, they do not take into account the details of the geometry of the device. Therefore, for numerical simulations the linear and nonlinear spring constants (*k* and *k*_*n*_, respectively) were determined from more accurate FEA analysis using COMSOL Multiphysics. The electromagnetic coupling factor (*γ*(z) = *dϕ*/*dz*) is determined from FEA analysis using Ansoft Maxwell, where *ϕ* denotes the magnetic flux linkage. The electromagnetic coupling factor (*γ*) is found to be a polynomial function (*γ*(*z*) = *B*_1_ + 3*B*_3_*z*^2^ + 5*B*_5_*z*^4^, where *B*_1_ = 5.94 Wb/m; *B*_3_ = −3.26 × 10^5^ Wb/m^3^; *B*_5_ = 5.39 × 10^9^ Wb/m^5^) dependent upon the deflection (*z*). These parameter values determining the magnetic flux distribution across the coil were put in the ODE (9) and solved numerically in Matlab. The parameters used in the numerical simulation are given in the Table 1.

### Fabrication of the nonlinear VEH devices

The device structure is made of FR4 (PCB) material using laser micromachining process. The Young’s modulus of FR4 is relatively low (18–23 GPa) in comparison to other commonly used metallic alloys, which implies applicability in low frequency vibrations. In addition, FR4 is lightweight (1.85 g/cc) yet mechanically robust with high yield strength (440–650 MPa), and capable of sustaining long-term oscillatory stress.

Furthermore, FR4 is relatively tolerant to variations in environmental conditions, such as temperature and humidity, which makes it an excellent material to use in unpredictable and potentially hazardous conditions. In the course of the experiments the FR4 based beam structure has been subject to more than 10 million cycles of oscillation, without any damage or degradation in performance, which validates the long-term reliability of the material for VEH applications. The bistable-quartic spring design has been laser-micromachined from FR4 sheets of thickness 0.2 mm and 0.5 mm. Four NdFeB magnets (8 mm × 4 mm × 2 mm (↑), the ‘↑’ denotes the magnetization axis direction) are arranged on both sides of a slot in the device structure in such a way that a high flux gradient is created within the slot. Furthermore, two high permeability soft magnetic steel blocks (8 mm × 8 mm × 1.6 mm) are used on both sides of the magnet assembly to intensify the magnetic flux in the slot. A coil wound using 30 μm diameter enameled copper wire (6.5 mm outer diameter, 1.15 mm inner diameter, 1 mm thickness, 2500 turns, 720 Ω resistance) is placed at the midpoint of the slot within the high flux gradient magnet assembly. The entire device is supported on four cylindrical nylon spacers, two of which are attached at the fixed end. The other two spacers are attached to the mid-points of two longitudinal beams which are carved on each side of the device structure. It was found that when the mass (magnet assembly) oscillates vertically, a part of the two beams are subject to bending as well as longitudinal stretching deformation, which introduce a nonlinear restoring force dependent upon the third power of the displacement. The middle portion of the beam supporting the magnets is designed to be thicker (0.5 mm) in comparison to the stretchable sections (0.2 mm) of the beam. Another NdFeB magnet (4 mm × 2 mm (↑) × 1 mm) with polarity along the longitudinal direction is attached at the tip of the device structure. In order to incorporate bistability, an additional NdFeB magnet (4 mm × 2 mm (↑) × 1 mm) is mounted on a micro-positioning stage and positioned in opposite polarity to the tip magnet. This arrangement exerts adjustable repulsive force on the tip magnet and induces bistable nonlinearity into the system dynamics.

### Experimental set-up for characterization

The base of the fabricated VEH prototypes was attached to a Brüel & Kjær shaker (LDS V455) with accelerometer (DeltaTron Type 4517-002) feedback. The shaker was controlled by an LDS Comet vibration controller and the output from the controller was amplified using a power amplifier (LDS PA 1000 L) before feeding to the shaker. Different vibrational accelerations (0.02–1.6 g) with a frequency range of 10 Hz to 90 Hz in both forward and reverse frequency sweep (sweep rate of 0.333 Hz/s) was used to excite the device. The band-limited random vibration test was performed using vibration that generated a flat power spectral density (PSD) profile over the frequency band (10–110 Hz). The output time traces of the VEH device and the shaker were recorded using a data acquisition system (Picoscope 3000 series). The external repulsive magnet was mounted onto a micro-positioning stage (Newport) to exercise precise control over the gap between the repulsively positioned magnets. A variable resistance box (Centrad) was used to vary the resistive load across the energy harvester output terminals.

## Additional Information

**How to cite this article**: Podder, P. *et al*. Influence of combined fundamental potentials in a nonlinear vibration energy harvester. *Sci. Rep.* **6**, 37292; doi: 10.1038/srep37292 (2016).

**Publisher’s note:** Springer Nature remains neutral with regard to jurisdictional claims in published maps and institutional affiliations.

## Figures and Tables

**Table 1:** Parameters used in the numerical simulations.

Parameters	Symbols	Value	Unit
Elasticity modulus (Young’s modulus) of FR4	*E*	19 × 10^9^	Pa
Stretchable beam thickness	*t*_*S*_	2 × 10^−4^	m
Stretchable beam width	*w*_*S*_	2 × 10^−3^	m
Thick beam thickness	*t*_*R*_	5 × 10^−4^	m
Thick beam width	*w*_*R*_	4 × 10^−3^	m
Thick beam length	*l*	20 × 10^−3^	m
Equivalent mass	*M*	3.56 × 10^−3^	kg
Linear spring coefficient	*k*	440	N/m
Nonlinear spring coefficient	*k*_*n*_	5.88 × 10^8^	N/m^3^
Dipole moments of magnets in bistable configuration	*m*_*1*_*, m*_*2*_	12.64 × 10^−3^	A·m^2^
Gap distance between bistable magnets	*d*	variable	m
Mechanical damping coefficient	*ξ*	0.012	—
Inductance of the coil	*L*	3.425 × 10^−3^	H
Resistance of the coil	*R*_*Coil*_	710	Ohm
Load resistance	*R*_*Load*_	3000	Ohm
